# Von-Meyenburg Complex: A Case of Elevated Gamma-GT

**DOI:** 10.7759/cureus.71347

**Published:** 2024-10-13

**Authors:** José Diogo Martins, Manuel Ferreira, Francisco Teixeira da Silva

**Affiliations:** 1 Internal Medicine, Unidade Local de Saúde do Alto Minho, Viana do Castelo, PRT

**Keywords:** biliary hamartomas, gamma-glutamyl transferase (ggt), genetic predisposition, hepatobiliary contrast mri, hepatocellular carcinoma (hcc), liver imaging, liver lesions, non-alcoholic fatty liver disease (nafld), polycystic kidney and hepatic disease 1 (pkhd1), von-meyenburg complex

## Abstract

The Von-Meyenburg complex, also known as biliary hamartomas, are benign malformations of the intrahepatic bile ducts. Typically identified incidentally, these lesions are generally asymptomatic and have no pathological significance. However, their imaging characteristics can mimic malignancy, and they possess a rare potential for progression to hepatocellular carcinoma. Familial clusters suggest a potential genetic basis, with the polycystic kidney and hepatic disease 1 (PKHD1) gene most commonly associated.

## Introduction

Firstly described by Hanns von Meyenburg in 1918, the Von-Meyenburg complexes (VMC) comprise benign biliary hamartomas characterized by multiple small hepatic nodules. These nodules, covered by fibrous stroma, are typically detected incidentally during imaging studies and are generally asymptomatic, with prevalence estimates ranging from 0.6% to 5.6% in autopsy studies [1, 2]. Despite their benign nature, their imaging appearance can suggest malignancy, and in rare cases, they may progress to hepatocellular carcinoma [3]. Familial clusters indicate a genetic predisposition, with the polycystic kidney and hepatic disease 1 (PKHD1) gene being frequently implicated [4].

## Case presentation

A 55-year-old caucasian woman presented to the emergency department with complaints of nausea and mild diffuse abdominal pain for the previous 15 days. There were no alleviating or aggravating factors. A detailed medical history revealed the patient’s use of herbal products. She denied other accompanying factors such as diarrhea, vomiting, fever, or changes in skin and mucosal coloration.

Her previous medical history included depression, treated with trazodone (150 mg at night) and Diazepam (5 mg in the afternoon and evening), grade 1 obesity (body mass index 32 kg/m2), and obstructive sleep apnea treated with automatic positive airway pressure (APAP). She used herbal products but denied consumption of teas, tobacco, or illicit drugs, and reported occasional alcohol consumption (20 g/day).

On physical examination, she had normal skin and mucosal coloration, neither cyanosis nor jaundice and a soft, non-tender, depressible abdomen with no palpable masses or organomegaly. 

Laboratory tests showed elevated gamma-glutamyl transferase (GGT) at 103 U/L (reference range <38 U/L), with normal alkaline phosphatase, transaminases, and bilirubin levels. Hemoglobin, leukocyte, and platelet counts, renal function, and coagulation tests were normal. C-reactive protein (CRP) was also negative (Table 1).

**Table 1:** Initial Laboratory Results The initial laboratory results for the patient, highlighted an elevated gamma-glutamyl transferase (GGT) level and other parameters within normal ranges.

Test	Result	Reference Value
Gamma-Glutamyl Transferase (GGT)	103 U/L	<38 U/L
Alkaline Phosphatase	71 U/L	30-120 U/L
Aspartate Aminotransferase (AST)	28 U/L	8-35 U/L
Alanine Aminotransferase (ALT)	34 U/L	7-45 U/L
Total Bilirubin	0.59 mg/dL	0.3-1.2 mg/dL
Direct Bilirubin	0.3 mg/dL	<0.5 mg/dL
Hemoglobin	15.4 g/dL	11.8-15.8 g/dL
Leukocytes	5.8 x 10⁹/L	4.0-10.0 x 10⁹/L
Platelets	164 x 10⁶/L	150-400 x 10⁶/L
Creatinine	0.76 mg/dL	0.6-1.0 mg/dL
Estimated Glomerular Filtration Rate (eGFR)	88 mL/min/1.73 m²	(MDRD formula)
Prothrombin Time (PT)	11.9 seconds	10.4-13.6 seconds
Activated Partial Thromboplastin Time (aPTT)	35.2 seconds	31.4-43.8 seconds

**Figure 1:**
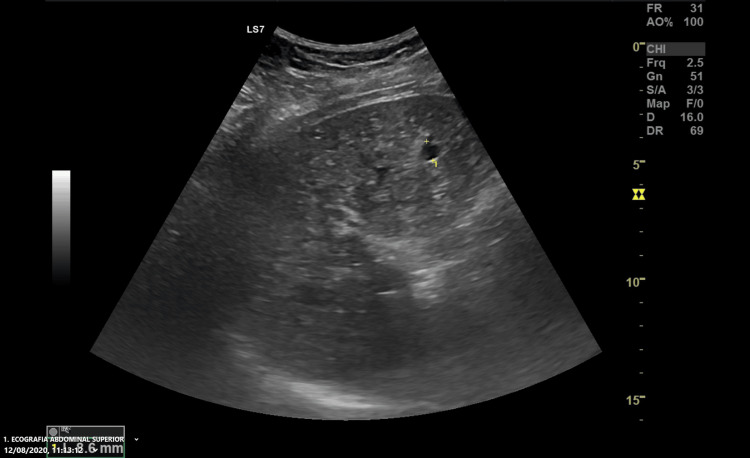
Abdominal Ultrasound Image Abdominal ultrasound revealing a liver with diffusely heterogeneous parenchyma and multiple indeterminate hypoechoic foci (the biggest one in this image measured and labeled with the number 1). This initial imaging study raised the suspicion of Von-Meyenburg complexes.

The abdominal ultrasound revealed a normal-sized liver with diffusely heterogeneous parenchyma, multiple indeterminate hyperechoic foci, and simple biliary cysts (Figure 1). There were no abnormalities in the gallbladder, intrahepatic, or extrahepatic bile ducts, pancreas, spleen, kidneys, or bladder. She was discharged with a referral to an Internal Medicine external consult and an indication to stop all non-prescription medications.

At the follow-up, she had discontinued herbal products and no longer had abdominal symptoms. Laboratory re-evaluation (Table 2) showed persistent gamma-glutamyl transferase (GGT) elevation (105 U/L) with otherwise normal hepatic and coagulation profiles, stable hemogram, and renal function, normal thyroid function, and no iron overload. She had mild dyslipidemia with total cholesterol of 195 mg/dL, low-density lipoproteins (LDL) of 134 mg/dL, and high-density lipoproteins (HDL) of 37 mg/dL. The infectious workup was negative for HIV, hepatitis B, and C, but indicated previous cytomegalovirus (CMV) and Epstein-Barr virus (EBV) infections. Immunological tests, including alpha-1 antitrypsin, ceruloplasmin, immunoglobulins, and hepatic autoimmune summary studies, were unremarkable.

**Table 2:** Follow-Up Laboratory Results The follow-up laboratory results, indicating persistent elevation of gamma-glutamyl transferase (GGT) and mild dyslipidemia, while other parameters remain within normal ranges. LDL:  low-density lipoproteins, HDL: high-density lipoproteins.

Test	Result	Reference Value
Gamma-Glutamyl Transferase (GGT)	105 U/L	<38 U/L
Thyroid-Stimulating Hormone (TSH)	1.66 µIU/mL	0.35-4.94 µIU/mL
Free Thyroxine (T4)	0.88 ng/dL	0.70-1.48 ng/dL
Iron	100 ng/dL	70-180 ng/dL
Transferrin	219 mg/dL	180-382 mg/dL
Ferritin	43.3 ng/mL	4.63-204.0 ng/mL
Total Iron-Binding Capacity (TIBC)	219 µg/dL	250-425 µg/dL
Total Cholesterol	195 mg/dL	<200 mg/dL
LDL Cholesterol	134 mg/dL	<100 mg/dL
HDL Cholesterol	37 mg/dL	>60 mg/dL

**Figure 2:**
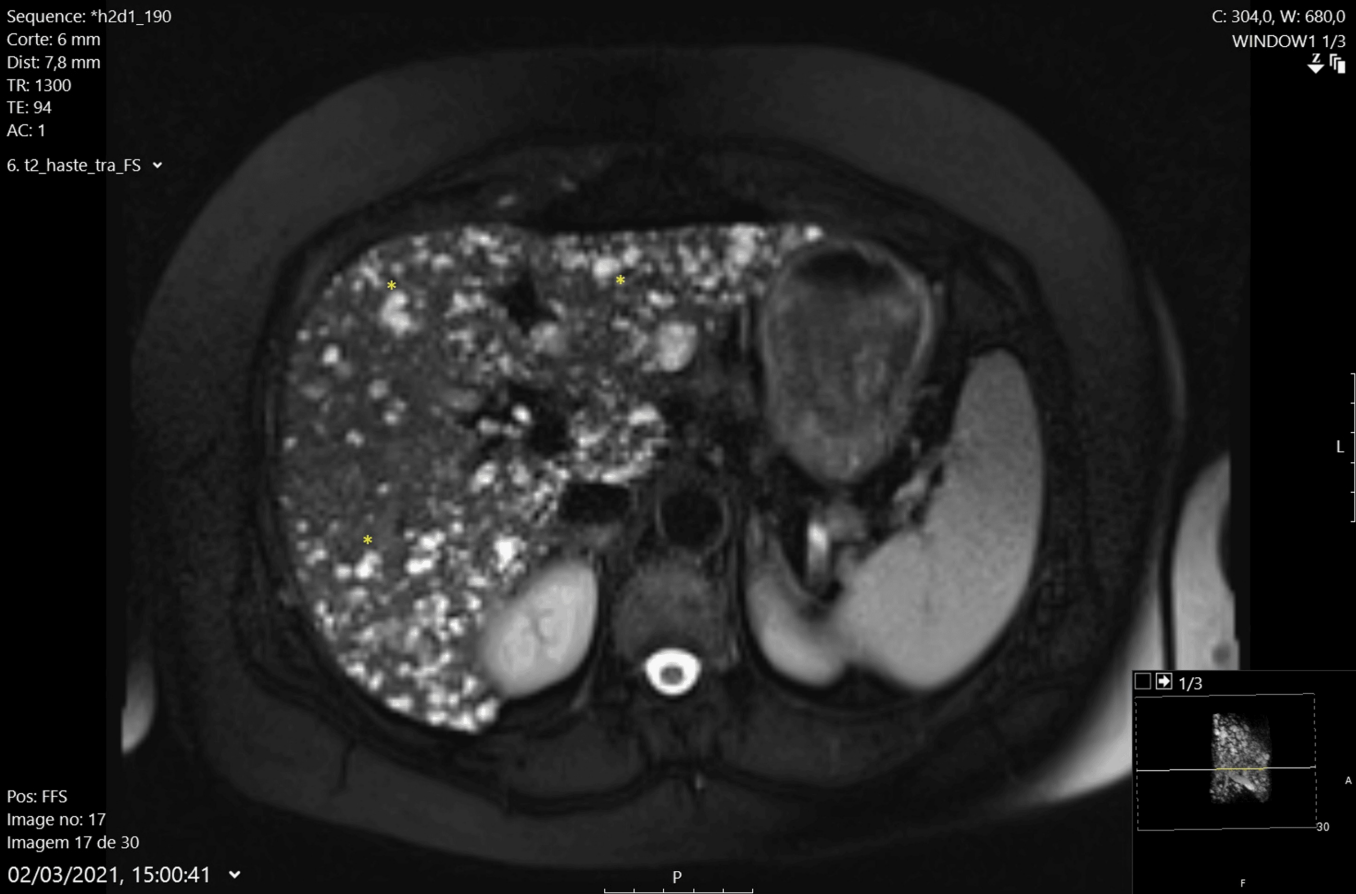
Axial MRI View of T2 Hyperintense Foci Axial MRI view displaying numerous T2 hyperintense lesions within the liver, indicative of Von-Meyenburg complexes (*). The image demonstrates the extensive distribution of these benign biliary hamartomas (*).

**Figure 3:**
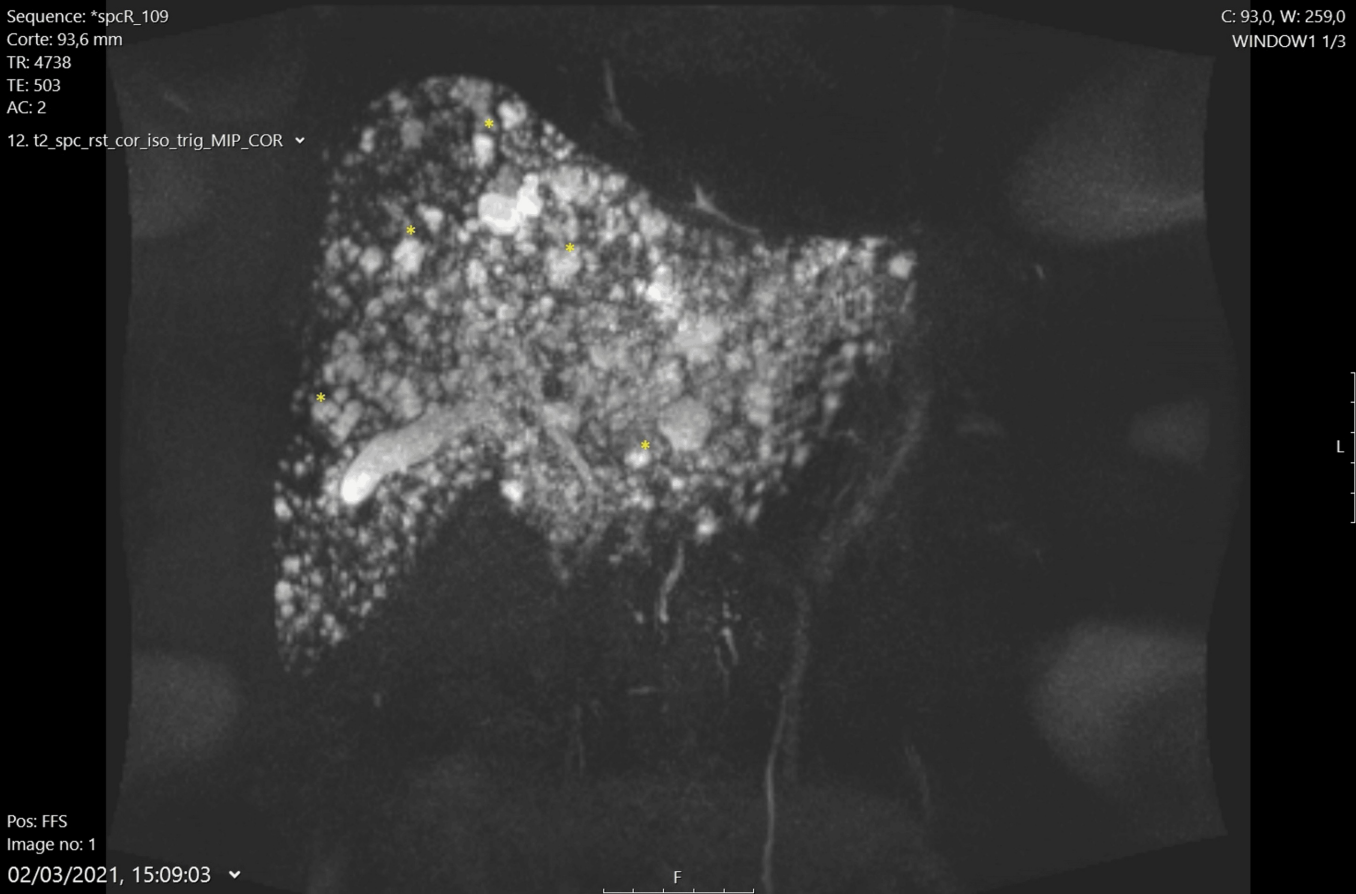
Abdominal MRI with T2 Sequence Abdominal MRI using T2-weighted sequence, illustrating numerous hyperintense foci typical of Von-Meyenburg complexes. The image provides a clear depiction of the benign nature of these biliary hamartomas.

Abdominal MRI with hepatobiliary-specific contrast (gadoxetate) confirmed a normal-sized liver with numerous T2 hyperintense foci throughout the parenchyma, suggesting multiple biliary hamartomas (Von-Meyenburg complexes) (Figures 2, 3). Mild splenomegaly was also noted. There were no dilated bile ducts or abnormalities in the gallbladder, pancreas, adrenal glands, or kidneys. No free fluid or abdominal adenopathies were observed. A liver biopsy was deemed unnecessary, and the patient was scheduled for continued clinical, laboratory, and imaging follow-up in the outpatient clinic.

## Discussion

While typically benign and asymptomatic, VMC can present significant diagnostic challenges due to its imaging characteristics that closely resemble malignant hepatic lesions. The recognition of VMC is critical in clinical practice to avoid unnecessary invasive procedures and anxiety associated with a potential misdiagnosis of liver cancer. The VMC was initially described by Hanns von Meyenburg in 1918 and involves clusters of dilated bile ducts encased in fibrous stroma, manifesting as small nodules scattered throughout the liver. These are often detected incidentally during imaging studies conducted for unrelated reasons [1].

In this case, the patient presented with nonspecific gastrointestinal symptoms and elevated gamma-glutamyl transferase (GGT), leading to further diagnostic evaluation. While elevated GGT is common in various liver conditions, including non-alcoholic fatty liver disease (NAFLD) and biliary obstruction, it is not specific to VMC. Therefore, imaging plays a crucial role in the diagnostic process.

The ultrasound findings indicated multiple hyperechoic foci within diffusely heterogeneous liver parenchyma, accompanied by simple biliary cysts, which raised initial suspicion. MRI further clarified these findings, revealing numerous T2 hyperintense foci throughout the liver, characteristic of VMC. Hepatobiliary-specific contrast agents in MRI enhanced diagnostic accuracy by providing detailed visualization of bile duct abnormalities [2, 3].

A genetic predisposition for VMC is suggested by its association with polycystic liver disease and autosomal dominant polycystic kidney disease (ADPKD). The PKHD1 gene, implicated in various fibrocystic diseases, has been associated with VMC, highlighting the importance of a thorough family history in patients presenting with liver cysts or nodules [4].

Although Von Meyenburg complexes are generally considered to have no pathological significance, there have been rare documented cases of progression to hepatocellular carcinoma, with some studies estimating an occurrence in less than 1% of cases. This potential malignancy appears to be linked to chronic liver injury or genetic predisposition. Consequently, this highlights the importance of vigilant monitoring, as early detection of malignant transformation can significantly impact patient outcomes. Regular follow-up with imaging studies is recommended to observe any changes in lesion characteristics that might suggest malignancy [5-6].

Management of VMC primarily involves monitoring and avoiding unnecessary invasive procedures. In this case, the decision against a liver biopsy was appropriate due to the clear imaging findings and the patient's clinical stability. The patient was advised to discontinue herbal products, which, although not directly implicated in VMC, could contribute to liver enzyme abnormalities and complicate the clinical picture. Lifestyle modifications, including weight management and avoidance of hepatotoxic substances, are essential in patients with liver lesions. While VMC itself may not cause significant morbidity, associated liver conditions such as NAFLD or drug-induced liver injury can exacerbate liver dysfunction and warrant careful management. Additionally, antibiotic use should be reserved for situations where there is concomitant infection, particularly of the biliary tract, and ursodeoxycholic acid can be employed to reduce cholestasis when indicated [7].

## Conclusions

This case underscores the importance of recognizing VMC as a differential diagnosis in patients with elevated liver enzymes and abnormal liver imaging findings. Proper diagnosis and vigilant follow-up are essential to manage these benign lesions effectively and mitigate the rare risk of malignant transformation. The case highlights the value of a multidisciplinary approach in managing complex liver pathologies, ensuring accurate diagnosis, appropriate monitoring, and optimal patient care.
